# Hybrid time series and machine learning models for forecasting cardiovascular mortality in India: an age specific analysis

**DOI:** 10.1186/s12889-025-23318-7

**Published:** 2025-06-10

**Authors:** M Darshan Teja, G Mokesh Rayalu

**Affiliations:** Department of Mathematics, School of Advanced Sciences, Vellore Institute of Technology, Vellore, 632 014 India

**Keywords:** ARIMA, Cardiovascular, Error Measures, Forecasting, Machine Learning

## Abstract

Cardiovascular disease (CVD) is a primary cause of death in India, accounting for a significant portion of the global CVD burden. This study looks at statistics on heart disease mortality from the Institute for Health Metrics and Evaluation (IHME) from 1990 to 2021, divided into five age groups: 0–5, 6–15, 16–49, 50–69, and 70 + . We used both classic ARIMA and hybrid models that combined ARIMA with machine learning techniques such as Random Forest, Support Vector Machine (SVM), XGBoost, and GARCH to anticipate mortality trends. Model performance was assessed using the Root Mean Square Error (RMSE) and Mean Absolute Percentage Error (MAPE). Across several age groups, the ARIMA + SVM model outperformed standalone ARIMA in terms of accuracy, with RMSE improvements of up to 15.6%. The 70 + population has the greatest mortality rates, highlighting the urgent need for focused healthcare treatments. These hybrid models are valuable tools for healthcare legislators in developing preventative programs, allocating resources effectively, and prioritizing treatment for high-risk age groups, especially the elderly, since they improve forecasting accuracy and offer interpretive insights. Given India's growing cardiovascular disease load, our results highlight how predictive analytics may support data-driven public health planning.

## Introduction

Cardiovascular diseases (CVDs), also termed heart disorders or strokes, represent the primary cause of mortality worldwide, responsible for one in three deaths globally. The majority of premature heart disease and stroke cases, despite their severe consequences, are preventable. In 2010, the worldwide expenditure on CVDs was roughly USD 863 billion, with projections indicating an increase of 22% to USD 1,044 billion by 2030 [1]. Disturbingly, 80% of deaths attributable to cardiovascular disease transpire in low- and middle-income nations. In India, non-communicable diseases, such as CVDs, are responsible for 60% of adult mortality, with CVDs constituting more than a quarter (26%) of these deaths. Contributing factors encompass elevated tobacco usage (15% of the population), a yearly average alcohol intake of 4.3 L of pure alcohol per capita, and hypertension impacting 21.1% of the population [2]. Hypertension independently elevates the risk of myocardial infarction, heart failure, renal illness, and cerebrovascular accident, underscoring the imperative for efficient monitoring and preventive measures.

Using time series analysis along with ARIMA models and machine learning techniques is a good way to look at, predict, and understand trends in health-related data, like the number of people who get heart disease and the death rate. Auto-Regressive Integrated Moving Average (ARIMA) models are important for finding temporal trends and seasonality in time-series data as in jian et al., (2022) [3]. They also make it easier to make accurate predictions and help with policymaking and allocating resources. Adding machine learning methods like random forests, support vector machines (SVMs), and XGBoost to ARIMA models makes forecasting better by taking into account complex interactions and nonlinear correlations in the data Singh.P et al., (2021) [4]. Multiple domains have effectively utilized hybrid models to forecast disease outbreaks, monitor health trends, and enhance healthcare interventions.

Although ARIMA is a prevalent model for time series forecasting, it exhibits significant limitations when used with intricate health-related datasets. The ARIMA model is linear, which means it doesn't effectively capture the complex relationships influenced by changing factors like medical treatments, lifestyle changes, seasonal patterns, and policy shifts. Additionally, it mainly relies on past data and assumes that conditions remain stable, making it less effective at detecting sudden changes caused by outside events like pandemics or economic shifts. The predicted accuracy also tends to diminish over extended timeframes. This study uses ARIMA along with advanced machine learning methods Random Forest, XGBoost, and Support Vector Machine to address these problems, as they can understand complex patterns, handle many variables, and include outside factors. These combined models improve the accuracy of predictions and provide useful information for decision-makers to better allocate healthcare resources and create prevention programs for vulnerable groups.

These methods can detect emerging trends in heart disorders, evaluate the influence of risk variables, and guide preventative strategies, thereby decreasing mortality rates and healthcare expenses. This study utilizes time-series modeling and machine learning to deliver actionable information about the trends and future impacts of cardiovascular diseases in India.

This research paper aims to develop and assess a hybrid forecasting method that integrates ARIMA with sophisticated machine learning techniques to improve the precision and utility of disease prediction models. This project seeks to offer significant insights into the effects of diseases across different age groups and to enhance evidence-based public health interventions. The explicit aims are as follows:

- To construct a hybrid forecasting model based on ARIMA: This means combining ARIMA with machine learning models like random forests, SVM, XGBoost and Garch to make predictions more accurate and handle complex nonlinear interactions in health datasets.
- To evaluate the prevalence of diseases by age group: Examine the prediction results across different age demographics to identify patterns, especially among those who are more susceptible to heart illnesses and other chronic ailments.
- To assess the effectiveness of hybrid and standalone models: Compare how well hybrid ARIMA models work to traditional statistical methods and separate machine learning methods, focusing on how well they can help with making decisions about public health and allocating resources.

## Literature review

The literature has extensively examined the comparative efficacy of time series and machine learning models.

Yu-Tse-Tsan et al. (2022) found that LSTM exhibited greater accuracy than ARIMA, particularly with extended datasets. In a ten-year dataset, LSTM attained a lower RMSE of 517.0, in contrast to 720.2 in a five-year sample, underscoring its capacity to discern intricate patterns in extended temporal datasets [5]. Yanchun Pan et al. (2016) demonstrated the effectiveness of ARIMA in predicting epidemic diseases with real-world data from January to August 2014, with a forecasting accuracy of 92.1%. This highlights ARIMA's efficacy in managing linear temporal data for short-term predictions [6].

Moreover, Krishnamoorthy et al. (2024) established that ARIMA surpassed LSTM in forecasting glucose and cholesterol levels, with a 71.7% reduction in RMSE for glucose and a 50.3% reduction in RMSE for cholesterol [7]. The results show that LSTM is better at finding non-linear trends in datasets, while ARIMA is better at finding linear patterns and making short-term predictions, often getting more accurate results in these situations.

Ji Eun Kim et al. (2024) employed ARIMA and generalized linear models (GLM) to forecast the incidence of inflammatory bowel disease (IBD) in Korea from 2018 to 2048. Their research revealed a consistent rise in IBD cases, with notable influences of age, gender, and temporal factors on incidence trends, underscoring differences within particular age cohorts [8]. Haowei Ni et al. (2024) showed that deep learning models, such as LSTM and PatchTST, are better at predicting cardiovascular disease (CVD) than older methods like ARIMA and Prophet [9]. They did this by collecting complex patterns in heart rate data more accurately. In 2024, Sicheng Shu showed an improved ARIMA-GARCH hybrid model for finding heart disease and predicting RR intervals. This model worked better at keeping trends and handling short-term volatility. Collectively, this research highlights the increasing significance of sophisticated models in disease prediction, providing prospective advantages for patient treatment and health management [10].

Senthil Pandi et al. (2024) examined malaria epidemics, emphasizing the necessity for early identification and predictive methodologies during the rainy season, when infections escalate [11]. Their findings indicate that, although exponential regression is helpful for short-term forecasts, the ARIMA model provides greater accuracy for prompt public health interventions. Salem Mubarak Alzahran and Fathelrhman EL Guma (2024) evaluated the predictive capabilities of XGBoost, ARIMA, and SARIMA models for seasonal influenza cases in Saudi Arabia. Their findings indicated that XGBoost surpassed conventional models by effectively capturing intricate nonlinear patterns, resulting in superior accuracy and reduced error rates [12]. Vishwajeet Singh et al. (2024) examined the global issue of monkeypox (MPOX) by employing ARIMA and Random Forest models to predict the disease's dissemination in severely impacted nations. The study demonstrated that Random Forest surpassed ARIMA in the majority of instances, highlighting its efficacy in predicting disease transmission [13].

Senthil et al. [14] created a hybrid ARIMA-ELMAN-ANN model that used strong preprocessing, feature selection, and clustering to accurately find people with diabetes early 96.31% of the time. Rafael Garcia et al. [15] used ElasticNet and Random Forest models to compare vaccination and non-vaccination scenarios and found a 9.19% mortality reduction in the first vaccination year in Spain [15]. A time-series-based healthcare monitoring system using deep learning (TS-CNN) by Shambhu Bhardwaj et al. (2024) exhibited promising real-time patient data categorization outcomes [16]. Nosratallah Forghani and Mohamad Forouzanfar (2024) examined ARIMA, LSTM, and the state-of-the-art Temporal Fusion Transformer (TFT) for heart rate prediction using ECG signals. The TFT model had the lowest MAE and RMSE [17].

Matthew Oyeleye et al. (2022) used ARIMA, linear regression, SVR, KNN, decision tree, random forest, and LSTM to predict heart rate (HR) using accelerometer-derived time-series data. ARIMA with walk-forward validation and linear regression scored well across all time durations, but other models excelled for predictions longer than one minute [18]. These findings suggest that time-series analysis and machine learning can reliably estimate HR using wearable devices. Fuad Ahmed Chyon et al. (2022) used the ARIMA model to predict early COVID-19 pandemic international infection trends. Using data from January 22 to April 7, 2020, they predicted 9.19 to 14.91 million confirmed cases worldwide in three months, assuming no containment measures change [19].

Chukwuka et al. (2024) emphasize the role of AI techniques, including machine learning and predictive modeling, in improving disease surveillance and public health interventions [20]. Cheng et al. (2020) present a simulation-driven optimization approach aimed at enhancing surveillance network architecture [21]. Kim et al. (2019) show that convolutional neural networks are better at predicting early death in pediatric ICU patients than traditional scoring systems [22]. Mahajan et al. (2023) recognize ensemble approaches, especially stacking, as exceptionally precise for illness prediction across many situations [23]. Mavaie et al. (2023) introduce a combined learning model that uses deep learning to pull out features and pairs it with simpler classifiers, leading to better results, especially for datasets that have a lot of features but not many examples [24].

For instance, Gaber and Singla (2025) showed the better performance of Random Forest Regressor in evaluating India's groundwater measurements using several regression models and assessment metrics, including RMSE and R-squared [25]. Mohamed (2025) underlined in the field of environmental sustainability the combination of waste-to-energy (WTE) technologies with evolutionary algorithms to maximize resource recovery and lower environmental impact [26]. Lewlisa et al. (2023) used a variety of ensemble and deep learning techniques for churn prediction; CNN and ANN models obtained up to 99% accuracy [27]. Alhussan et al. (2023) introduced a new method called DBERDTO to better classify diabetes in healthcare, achieving an accuracy of 98.6% and showing its importance through ANOVA and Wilcoxon tests [28]. Inspired by nature, El-Saymed et al. (2023) invented the Greylag Goose Optimization (GGO) method, demonstrated its efficacy on several datasets and engineering issues, and so surpassed current optimization strategies [29]. These investigations together reveal how well hybrid and intelligent algorithms increase system optimization, decision-making, and forecast accuracy in many different fields.

The analyzed research predominantly concentrates on either ARIMA and alternative time-series models or machine learning methods for disease prediction. Forecasting patterns related to COVID-19, diabetes, and cardiovascular diseases have widely employed ARIMA, demonstrating its efficacy in short-term predictions. At the same time, machine learning models such as LSTM, SVR, and XGBoost have shown great success in finding complex patterns and nonlinear correlations in data, especially over long periods of time or in datasets with a lot of variation.

This research uniquely mixes time-series models, such as ARIMA, with advanced machine learning approaches inside a hybrid analytical framework. This method leverages the benefits of ARIMA for trend analysis and machine learning models to identify nonlinear relationships, thereby improving the accuracy and reliability of disease predictions. This research integrates various approaches to offer a thorough solution for evaluating illness progression and facilitating effective public health treatments.

## Methods

### Data source

The data for this study was sourced from the Institute for Health Metrics and Evaluation (IHME) via the Global Burden of Disease (GBD) database (https://www.healthdata.org/research-analysis/gbd), covering the period from 1990 to 2021. The dataset examines death rates in India over five specific age categories: 0–5, 6–15, 16–49, 50–69, and 70 + . This extensive dataset, publicly accessible on the IHME GBD website, offers high-quality, complete data devoid of any missing values. This study focuses only on India to examine age-specific mortality trends and patterns associated with heart disease and other chronic illnesses, despite the GBD database containing worldwide data for other nations.

### ARIMA (Auto-regressive integrated moving average)

The statistical method known as ARIMA integrates autoregressive (AR), differencing (I), and moving average (MA) components for time series forecasting [30].

1 $${X}_{t}=c+{\vartheta }_{1}{X}_{t-1}+{\vartheta }_{2}{X}_{t-2}+\dots +{\vartheta }_{p}{X}_{t-p}-{\theta }_{1}{e}_{t-1}-{\theta }_{2}{e}_{t-2}-\dots -{\theta }_{q}{e}_{t-q}+{e}_{t}$$

where:

- ${X}_{t}:$ Actual Value at time t
- $$c:$$ Constant term
- ${\vartheta }_{1},{\vartheta }_{2},\dots \dots \dots ,{\vartheta }_{p}:$ Coefficients for the autoregressive terms
- ${\theta }_{1},{\theta }_{2},\dots \dots \dots ,{\theta }_{q}:$ Coefficients for the moving average terms
- ${e}_{t}:$ Random error at time t

### ARIMA with random forest (ARIMA + RF)

Random Forest (RF) is an excellent machine learning model for capturing nonlinear correlations in time-series data, whereas ARIMA falls short due to its linear assumptions. As a method that uses multiple decision trees, RF can understand complex relationships between different factors and is especially helpful when outside influences like environmental, behavioral, or demographic factors significantly affect patterns of cardiovascular deaths. Its capacity to handle multidimensional data makes it an excellent companion to ARIMA in hybrid forecasting frameworks [31].

2 $${X}_{t}={\widehat{{X}_{t}}}^{ARIMA}+{\widehat{{R}_{t}}}^{RF}$$

- ${X}_{t}:$ Actual Value at time t
- ${\widehat{{X}_{t}}}^{ARIMA}:$ Linear forecast from the ARIMA model
- ${R}_{t}={X}_{t}-{\widehat{{X}_{t}}}^{ARIMA}:$ Residuals from the ARIMA model
- ${\widehat{{R}_{t}}}^{RF}:$ Prediction of residuals using random forest.

### ARIMA with Support Vector Machine (ARIMA + SVM)

This methodology integrates ARIMA with support vector machines, which are proficient in modeling non-linear interactions. The ARIMA model forecasts the linear component of the series, whereas the SVM addresses the non-linear residuals [32]. ARIMA models depend exclusively on historical values and are constrained in their ability to manage high-dimensional or intricate data structures. On the other hand, Support Vector Machines (SVM) use kernel functions to change input data into higher dimensions, which helps them find patterns better, especially in messy or complex datasets. This renders SVM especially appropriate for predicting outcomes in structured health data characterized by both linear and nonlinear dependencies.

3 $${X}_{t}={\widehat{{X}_{t}}}^{ARIMA}+{\widehat{{R}_{t}}}^{SVM}$$

- ${X}_{t}:$ Actual Value at time t
- ${\widehat{{X}_{t}}}^{ARIMA}:$ Linear forecast from the ARIMA model
- ${R}_{t}={X}_{t}-{\widehat{{X}_{t}}}^{ARIMA}:$ Residuals from the ARIMA model
- ${\widehat{{R}_{t}}}^{SVM}:$ Prediction of residuals using SVM

### ARIMA with Extreme gradient boosting (ARIMA + XGB)

XGBoost is an ensemble learning technique based on decision trees. XGBoost uses the residuals from the ARIMA model to improve forecasting precision [33]. ARIMA frequently has difficulties in long-term forecasting due to its reliance on historical data and its limited capacity to adapt to changing patterns. XGBoost, a robust gradient boosting technique, adeptly manages feature importance, iteratively rectifies residual mistakes, and captures intricate connections within the data. Its efficacy in handling missing values and scalability with extensive datasets renders it a formidable option for improving forecasting precision, especially over prolonged timeframes.

4 $${X}_{t}={\widehat{{X}_{t}}}^{ARIMA}+{\widehat{{R}_{t}}}^{XGB}$$

- ${X}_{t}:$ Actual Value at time t
- ${\widehat{{X}_{t}}}^{ARIMA}:$ Linear forecast from the ARIMA model
- ${R}_{t}={X}_{t}-{\widehat{{X}_{t}}}^{ARIMA}:$ Residuals from the ARIMA model
- ${\widehat{{R}_{t}}}^{XGB}:$ Prediction of residuals using XGB

### ARIMA with generalized autoregressive conditional heteroskedasticity (ARIMA + GARCH)

The ARIMA + GARCH hybrid model is a bifurcated time series forecasting methodology that integrates the advantages of ARIMA (AutoRegressive Integrated Moving Average) for delineating the linear and mean characteristics of a time series with GARCH (Generalized Autoregressive Conditional Heteroskedasticity) for addressing volatility clustering and time-dependent variance (heteroskedasticity) in the residuals [34]. This approach is especially beneficial for real-world datasets, such as financial or health-related data, when both linear trends and conditional variance patterns, such as volatility or shocks, are present.

Stage 1: ARIMA for mean modelling

An ARIMA(p,d,q) model is first fit to the data:

5 $$\varphi (B{)(1-B)}^{d}{y}_{t}=\theta (B){\epsilon }_{t}$$

$\varphi \left(B\right)$: Autoregressive (AR) polynomial

- $\theta \left(B\right)$: Moving Average (MA) polynomial
- *d*: Degree of differencing
- B: Backshift operator, $$B{y}_{t}={y}_{t-1}$$
- ${\epsilon }_{t}$: White noise residuals

Stage 2: GARCH for volatility modeling

The residuals ${\epsilon }_{t}$ from the ARIMA model are then modeled with GARCH (r,s):

6 $${\epsilon }_{t}={z}_{t}\sqrt{{h}_{t}}, {z}_{t}\sim N(\text{0,1})$$

7 $${h}_{t}=w+\sum_{i=1}^{r}{\alpha }_{i}{\epsilon }_{t-i}^{2}+\sum_{j=1}^{s}{\beta }_{j}{h}_{t-j}$$

${h}_{t}$: Conditional Variance at time t

- W > 0, $${\alpha }_{i}\ge 0, {\beta }_{j}\ge 0$$

### RMSE (Root Mean Square error)

RMSE quantifies the square root of the mean of the squared deviations between expected and actual values [35, 36].

8 $$RMSE=\sqrt{\frac{1}{n}\sum_{i=1}^{n}{({y}_{i}-\widehat{{y}_{i}})}^{2}}$$

where ${y}_{i}:$ Actual Value, $\widehat{{y}_{i}}:$ Predicted Value, $n:$ Number of observations.

### MAPE (Mean Absolute percentage error)

MAPE calculates the average percentage error between actual and predicted values.

9 $$MAPE=\frac{1}{n}\sum_{i=1}^{n}\left|\frac{{y}_{i}-\widehat{{y}_{i}}}{{y}_{i}}\right|\times 100$$

where ${y}_{i}:$ Actual Value, $\widehat{{y}_{i}}:$ Predicted Value.

### AIC (Akaike Information Criterion)

AIC assesses the quality of a statistical model by reconciling goodness-of-fit with model complexity.

10 $$AIC=2k-2ln\left(L\right)$$

where:

k: Number of parameters in the model.

L: Maximum likelihood of the model.

### BIC (Bayesian Information Criterion)

BIC resembles AIC but imposes a more stringent penalty on models with an increased number of parameters.

11 $$BIC=kIn\left(n\right)-2In\left(L\right)$$

where:

k: Number of parameters.

n: Number of observations.

Since the dataset only had yearly death data for five different age groups and didn't include outside factors or complex inputs, we could only look at one time series for each age group. Thus, traditional feature engineering strategies, such as multivariate inputs or lagged variables, proved ineffective. Rather, we ensured that each time series underwent stationarity testing and, if necessary, differencing. We submitted the model's residuals to machine learning models (RF, SVM, XGBoost, GARCH) trained to learn nonlinear patterns that ARIMA could not capture. We chose these models for structured data due to their interpretability, resistance to overfitting, and ability to explain complex linkages. While deep learning techniques such as LSTM and GRU can effectively represent lengthy sequences, we focused on computationally efficient models that prioritize interpretability. Deep learning approaches and exogenous factors for greater predictive performance may aid future research in expanding this effort.

## Results and discussion

The dataset classified about heart disease-related fatalities in India into distinct age brackets: 0–5, 6–15, 16–49, 50–69, and 70 + . The data transformed in each age group into a time series format to study trends and patterns across time. This modification facilitated the assessment of temporal dynamics and trend prediction. A vital aspect of time series analysis is ensuring stationarity, as the majority of time series models, including ARIMA, necessitate that the data maintains a consistent mean and variance throughout time [30]. The evaluated stationarity for each age group using the Augmented Dickey-Fuller (ADF) test.

Table 1 summarizes the results of the ADF test. The test yields the Dickey-Fuller test statistic, lag order, and associated p-value for each age cohort. The null hypothesis of the ADF test posits the existence of a unit root, indicating that the data is non-stationary. If the p-value is less than the significance level (often 0.05), the null hypothesis is rejected, signifying that the series is stationary.

**Table 1:** ADF test results for different age group

Age	Dickey-Fuller	Lag order	P-Value
0 to 5	-3.1608	3	0.05
6 to 15	-3.5562	3	0.04
16 to 49	-3.3165	3	0.05
50 to 69	-3.5068	3	0.01
70 +	-3.8805	3	0.01

All age groups have p-values at or below 0.05, suggesting that we can reject the null hypothesis of non-stationarity. This indicates that the time series data for each age group is steady, rendering it appropriate for ARIMA time-series modeling. The Dickey-Fuller test data demonstrate a pronounced inclination toward stationarity, especially in the older age cohorts (50–69 and 70 +), characterized by a notably low *p*-value (0.01). This discovery underscores the resilience of the time series data across all age demographics and establishes a basis for subsequent research and predictions.

### ACF, PACF and residuals analysis

In this Research produced the Autocorrelation Function (ACF) and Partial Autocorrelation Function (PACF) plots to conduct a more in-depth analysis of the time series data for each age group. These plots facilitate the identification of the correlation structure within the data and inform the selection of suitable parameters for the ARIMA model. The ACF looks at how well different sets of data are related over different time periods, while the PACF shows how an observation from the past is directly linked to the present value, without taking into account any intermediate delays.

**Fig. 1:**
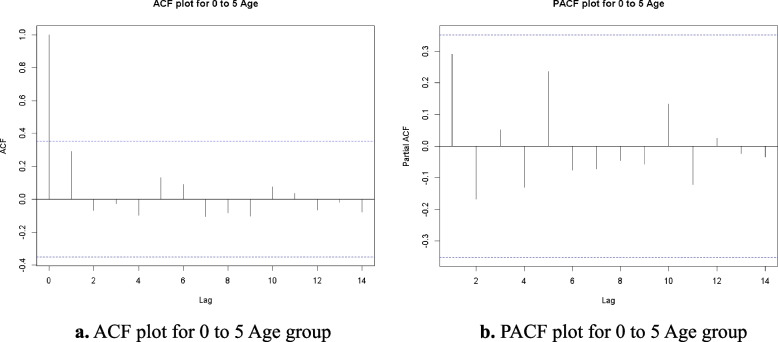
**a** ACF plot for 0 to 5 Age group. **b** PACF plot for 0 to 5 Age group

**Fig. 2:**
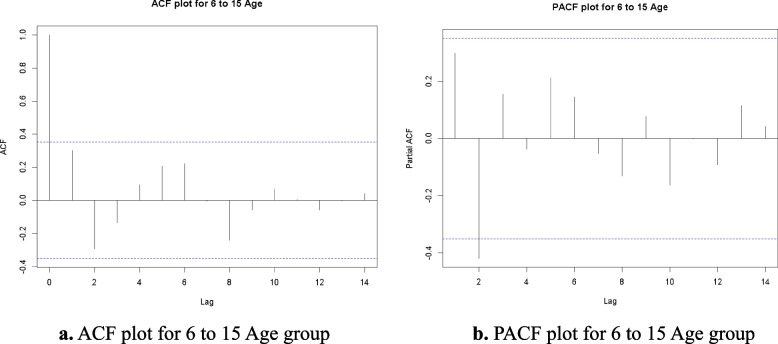
**a** ACF plot for 6 to 15 Age group. **b** PACF plot for 6 to 15 Age group

**Fig. 3:**
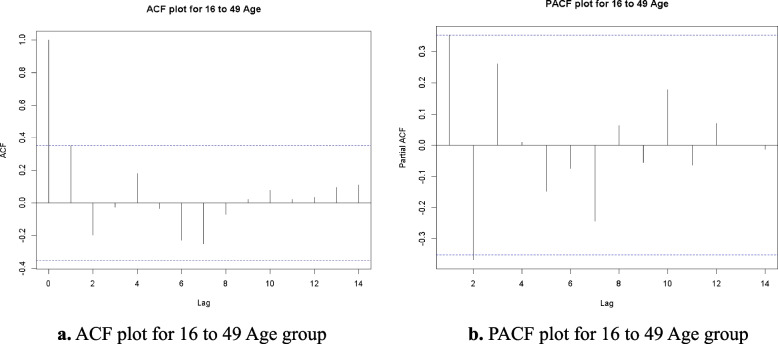
**a** ACF plot for 16 to 49 Age group. **b** PACF plot for 16 to 49 Age group

**Fig. 4:**
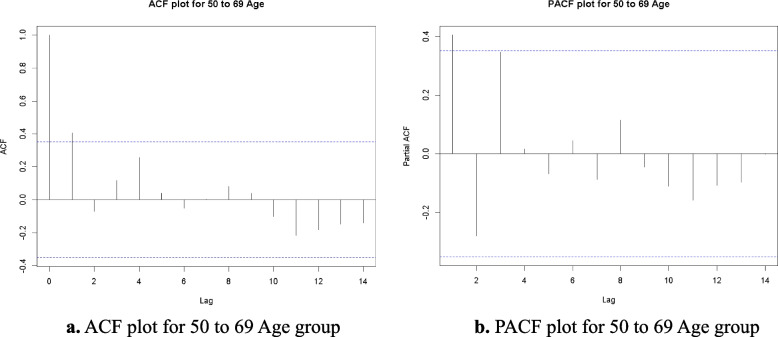
**a** ACF plot for 50 to 69 Age group. **b** PACF plot for 50 to 69 Age group

**Fig. 5:**
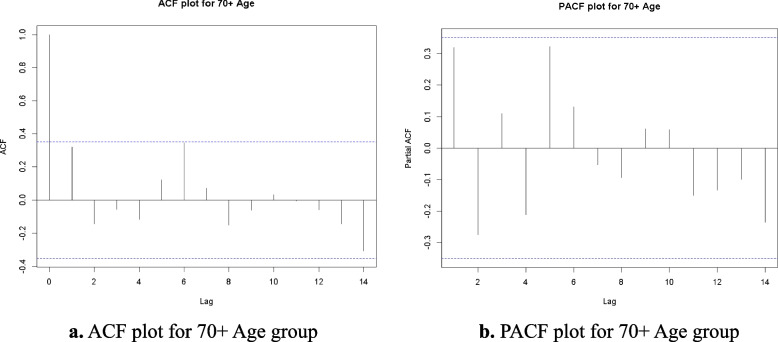
**a** ACF plot for 70 + Age group. **b** PACF plot for 70 + Age group

Figures 1a, 2, 3, 4 and 5 b illustrate the ACF and PACF plots for each age group: 0–5, 6–15, 16–49, 50–69, and 70 + . In every instance, the autocorrelation and partial autocorrelation values reside within the confidence intervals, denoted by the lower limit (LL) and upper limit (UL). This indicates that there are no significant lagged correlations beyond the boundaries of randomness, which suggests that the series does not exhibit significant seasonal or cyclical patterns.

**Fig. 6:**
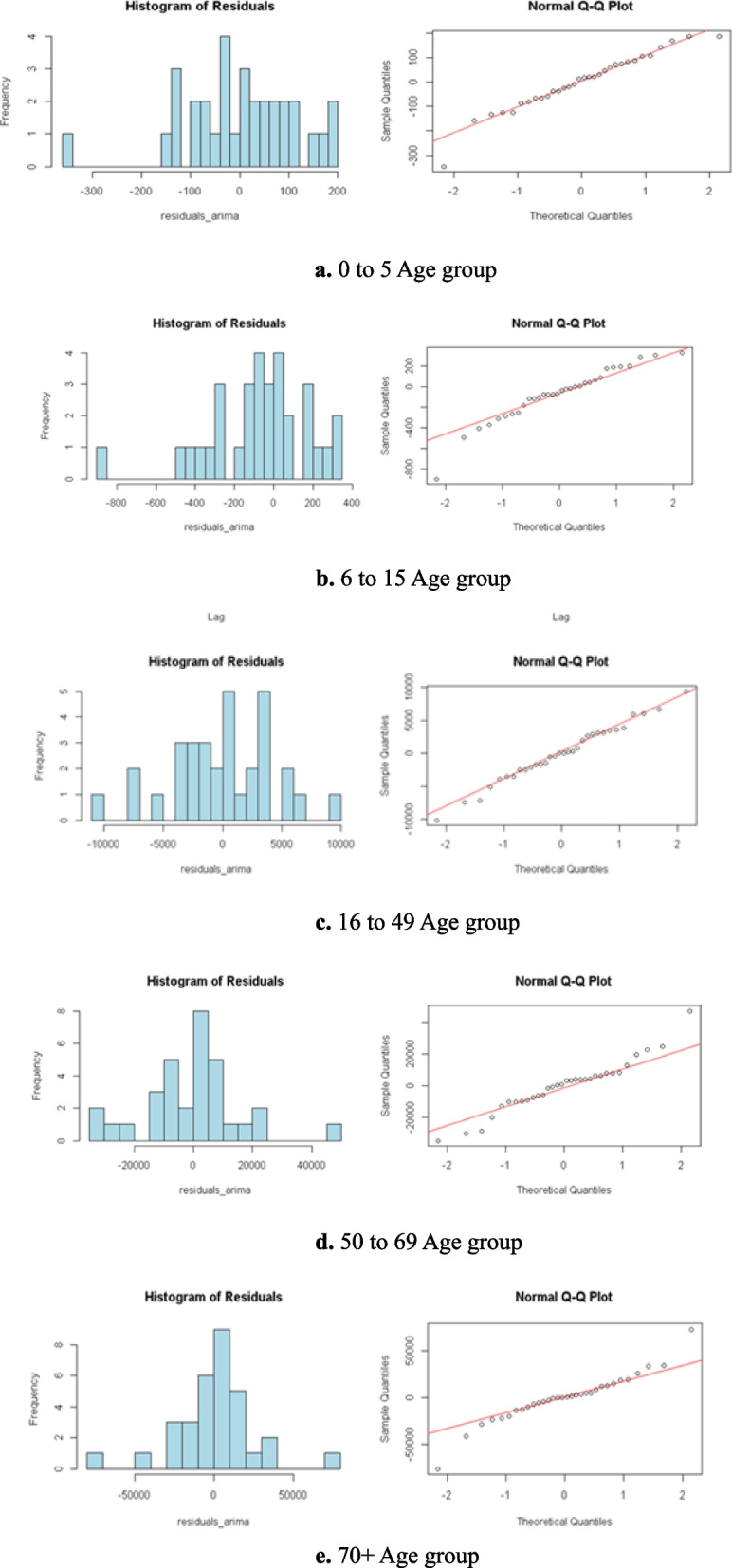
**a** 0 to 5 Age group. **b** 6 to 15 Age group. **c** 16 to 49 Age group. **d** 50 to 69 Age group. **e** 70 + Age group

Figure 6a to e provide a histogram and normal Q-Q plots of residuals for each age cohort. Across all age demographics, the Q-Q plots illustrate that most residual values are in proximity to the red reference line, signifying that the residuals are approximately normally distributed. This validates the residual normality assumption and affirms the suitability of ARIMA-based models for analyzing patterns of heart disease mortality across different age groups.

The results show that the time series data for all age groups can be used for ARIMA modeling because there are no significant autocorrelations or partial autocorrelations that are not taken into account within acceptable confidence levels. This conclusion underscores the dataset's robustness and endorses the use of time-series forecasting methods for heart disease mortality trends across various age demographics.

### ARIMA analysis

The utilized ARIMA model on the time series data for each age cohort (0–5, 6–15, 16–49, 50–69, and 70 +) to determine the optimal model for predicting heart disease mortality trends. The AIC determined the model selection for each age group, favoring models with the lowest AIC values. The table below presents the chosen ARIMA models and their associated parameters, including the Moving Average (MA1) coefficient, drift, log-likelihood, AIC, corrected AIC (AICC), and BIC.

It was found that the ARIMA (0,1,1) and ARIMA (0,1,1) with drift models fit most age groups the best, as shown by their lower AIC and log-likelihood values in Table 2. The drift parameter was important in the models for age groups 0–5, 16–49, 50–69, and 70 + , which means that the time series data for these groups consistently showed an upward or downward trend. The MA1 coefficient exhibited variability among age groups while consistently capturing short-term associations efficiently.

**Table 2:** ARIMA model selection for different age group and their related results

Age group	ARIMA Model	MA1	drift	Log likelihood	AIC	AICC	BIC
0–5	(0,1,1) with drift	0.4438	-293.113	-190.62	387.25	388.14	391.55
6–15	(0,1,1)	0.6724	-	-217.56	439.12	439.55	441.99
16–49	(0,1,1) with drift	0.5536	3368.132	-303.8	613.6	614.49	617.91
50–69	(0,1,1) with drift	0.6305	18,114.304	-345.04	696.07	696.96	700.37
70 +	(0,1,1) with drift	0.6449	32,157.314	-358.68	723.37	724.25	727.67

The age group 6–15 was modeled without drift, as incorporating a drift term did not substantially enhance the model's fit. This indicates that the trend component for this age group is either less pronounced or absent compared to the other categories. The chosen models illustrate ARIMA's capacity to precisely represent the dynamics of heart disease mortality across several age groups, establishing a basis for exact forecasting and trend analysis.

We used the Breusch-Pagan (BP) test to determine whether heteroscedasticity existed in the residuals of the ARIMA models across different age groups. The results showed in Table 3 that all five age groups 0–5, 6–15, 16–49, 50–69, and 70 + had p-values higher than the usual cutoff of 0.05, meaning there was no strong evidence of heteroscedasticity. This means that the ARIMA models' residuals have constant variance (homoscedasticity), which supports the validity of the modeling assumptions and the durability of time series models for forecasting trends in heart disease mortality.

**Table 3:** Breusch-Pagan test results for Heteroscedasticity to different Age groups

Age Group	BP Statistic	Df	p-value	Heteroscedasticity
0–5	1.7715	1	0.1832	No
6–15	1.4622	1	0.2266	No
16–49	2.3631	1	0.1242	No
50–69	1.5898	1	0.2074	No
70 +	2.6854	1	0.1013	No

### ARIMA with hybrid models

Once we knew which ARIMA models worked best for each age group, we combined ARIMA with machine learning techniques like random forests, support vector machines, XGBoost and GARCH to make hybrid models. The hybrid method combines the best features of ARIMA for modeling linear and temporal dependencies with the power of machine learning models to find complex patterns and correlations in the data that don't follow a straight line. This methodology seeks to enhance the forecast of heart disease mortality among various age demographics.

We used the residuals from the ARIMA model as input for the machine learning models in each age group (0–5, 6–15, 16–49, 50–69, and 70 +). These residuals signify inexplicable fluctuations subsequent to the use of the ARIMA model. We trained the machine learning models to predict the residuals, then incorporated them into the ARIMA forecasts to produce the final hybrid forecasts.

Future value forecasts were produced using both ARIMA and hybrid ARIMA-ML models. The efficacy of each hybrid model was assessed using metrics like RMSE and MAPE. The projected values for each age demographic are included in the accompanying table and shown in graphics.

#### Error Metrics Performances

The predictive efficacy of standalone ARIMA and hybrid models (ARIMA + RF, ARIMA + SVM, ARIMA + XGBoost, and ARIMA + GARCH) was assessed across various age demographics utilizing RMSE and MAPE are shown in Table 4. In the 0–5 age range, the ARIMA + GARCH model achieved the best performance with lowest RMSE (255.8) and MAPE (5.89), slightly outperforming standalone ARIMA indicating its capability to model volatility in early-age mortality. Comparing ARIMA to hybrid models, hybrid models did better with kids ages 6 to 15, with ARIMA + SVM being the most effective (RMSE: 110.57, MAPE: 4.35), closely followed by ARIMA + RF. In the 16–49 age demographic, ARIMA + SVM attained the lowest RMSE (326.90) and MAPE (0.16), indicating its greater accuracy compared to alternative models. Within the 50–69 age demographic, the ARIMA + XGBoost model had the highest accuracy, evidenced by the lowest RMSE (239.72) and MAPE (0.05), closely succeeded by ARIMA + SVM. In the 70 + age range, ARIMA + SVM surpassed all other models, achieving the lowest RMSE (25,254.45) and MAPE (1.77), while ARIMA + RF also showed enhancements compared to the standalone ARIMA model. Over all the results affirm that hybrid models, particularly those integrating SVM and XGBoost with ARIMA, substantially enhance predictive accuracy, while GARCH models offer moderate improvements primarily in scenarios with time-varying variance.

**Table 4:** Evaluation of forecasting precision among age demographics utilizing standalone ARIMA and hybrid models (ARIMA + RF, SVM, XGBoost, GARCH) According to RMSE and MAPE Metrics

Models	0–5		6–15		16–49		50–69		70 +	
	RMSE	MAPE	RMSE	MAPE	RMSE	MAPE	RMSE	MAPE	RMSE	MAPE
ARIMA	266.5	6.33	258.2	10.6	500.0	0.95	815.8	0.36	27,658.8	1.99
ARIMA with RF	466.5	11.95	121.6	5.03	615.2	1.06	795.9	0.21	26,844.8	1.88
ARIMA with SVM	364.2	9.33	110.5	4.35	326.9	0.16	417.4	0.07	25,254.4	1.77
ARIMA with XGB	606.4	15.5	182.0	7.57	670.0	2.16	239.7	0.05	28,575.4	2.00
ARIMA with GARCH	255.8	5.89	263.3	11.5	671.8	2.17	878.6	0.75	29,387.6	2.15

#### Statistical Significant testing for forecasting error

The paired t-test and the Wilcoxon signed-rank test were used to statistically evaluate the performance differences between the hybrid models, using RMSE values from cross-validation results. The comparison of ARIMA + RF and ARIMA + XGBoost produced a p-value of 0.598 (t-test) and 0.9219 (Wilcoxon test), signifying no statistically significant difference in performance. The p-values of 0.179 (t-test) and 0.2324 (Wilcoxon test) indicated no statistically significant enhancement between ARIMA + SVM and ARIMA + XGBoost. The statistics show that even though the models perform differently, these differences are not statistically important at the 0.05 level, likely due to a small sample size or similar performance among the models. The ARIMA + GARCH model is aimed at understanding volatility and isn't made to minimize RMSE during cross-validation, so it was left out of this statistical test to keep the methods consistent.

#### Forecasting results for all Age groups

**Fig. 7:**
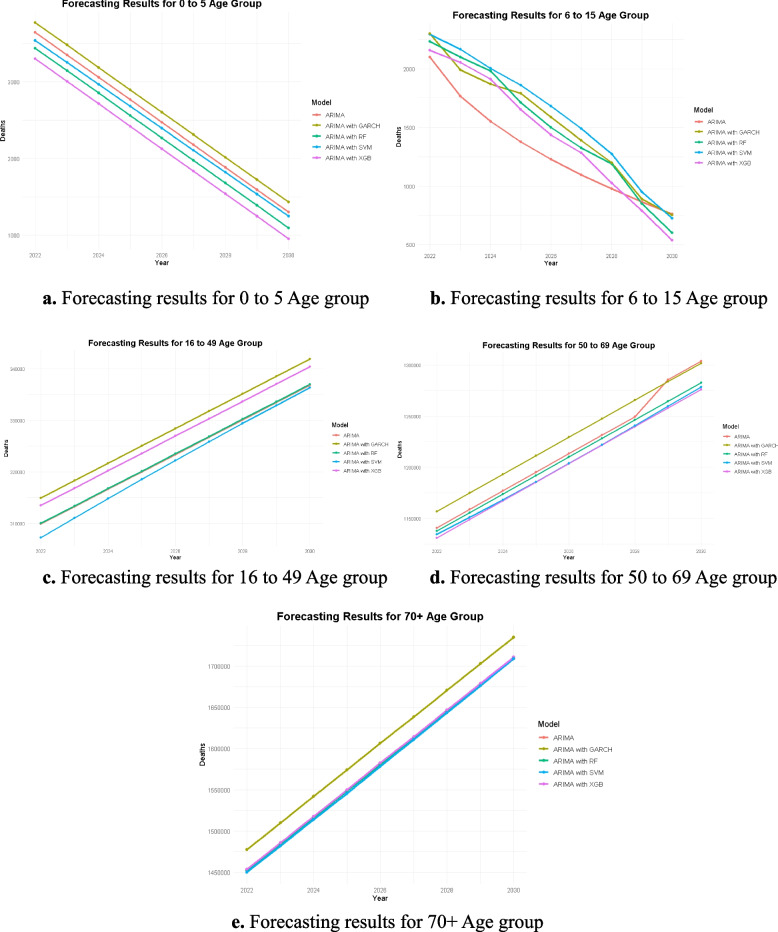
**a** Forecasting results for 0 to 5 Age group. **b** Forecasting results for 6 to 15 Age group. **c** Forecasting results for 16 to 49 Age group. **d** Forecasting results for 50 to 69 Age group. **e** Forecasting results for 70 + Age group

Tables 5, 6, 7, 8, 9 and Fig. 7a to e present the forecasting results for all age groups (0–5, 6–15, 16–49, 50–69, and 70 +) using standalone ARIMA and hybrid models (ARIMA + RF, ARIMA + SVM, ARIMA + XGBoost, and ARIMA + GARCH), showcasing distinct trends and model performances across age categories. Tables 5 and 6 illustrate that the 0–5 and 6–15 age groups exhibit a steady decrease in projected deaths. The standalone ARIMA model demonstrates optimal performance for the youngest cohort, as depicted in Fig. 7a to b, whereas the ARIMA + SVM and ARIMA + RF models yield enhanced accuracy for the 6–15 age group. Tables 7 and 8 demonstrate that the models predict a steady increase in mortality rates in the 16–49 and 50–69 age groups, highlighting the growing influence of cardiovascular diseases in these groups, as shown in Fig. 7c to d. For these groups, ARIMA combined with SVM consistently demonstrated superior performance, yielding more precise forecasts with reduced error metrics. In the age group of 70 and up shown in Table 9, all models predict a significant rise in mortality, as shown in Fig. 7e. ARIMA + XGBoost predicts the biggest rise, while ARIMA + SVM gives the most stable and reasonable predictions.

**Table 5:** Different models forecasting value for 0 to 5 age group

0 to 5 Age					
Year	ARIMA	ARIMA with RF	ARIMA with XGB	ARIMA with SVM	ARIMA with GARCH
2022	3643	3437	3297	3539	3771
2023	3350	3144	3004	3251	3478
2024	3056	2851	2711	2963	3185
2025	2763	2558	2418	2677	2893
2026	2470	2265	2125	2392	2600
2027	2177	1972	1832	2106	2307
2028	1884	1678	1537	1820	2015
2029	1591	1385	1245	1533	1722
2030	1298	1092	952	1246	1430

**Table 6:** Different Models Forecasting value for 6 to 15 Age group

6 to 15 Age					
Year	ARIMA	ARIMA with RF	ARIMA with XGB	ARIMA with SVM	ARIMA with GARCH
2022	2098	2233	2158	2294	2300
2023	1767	2100	2055	2165	1989
2024	1551	1980	1910	2001	1868
2025	1379	1710	1650	1860	1789
2026	1230	1500	1436	1680	1586
2027	1097	1325	1284	1489	1390
2028	976	1189	1025	1275	1198
2029	865	850	789	950	890
2030	761	600	539	725	753

**Table 7:** Different models forecasting value for 16 to 49 Age group

16 to 49 Age					
Year	ARIMA	ARIMA with RF	ARIMA with XGB	ARIMA with SVM	ARIMA with GARCH
2022	309,916	310,048	313,495	307,287	314,976
2023	313,284	313,416	316,863	311,054	318,343
2024	316,652	316,784	320,231	314,822	321,711
2025	320,020	320,152	323,599	318,560	325,078
2026	323,388	323,520	326,968	322,248	328,446
2027	326,756	326,889	330,336	325,872	331,813
2028	330,124	330,257	333,704	329,430	335,181
2029	333,492	333,625	337,072	332,926	338,548
2030	336,861	336,993	340,440	336,368	341,916

**Table 8:** Different models forecasting value for 50 to 69 age group

50 to 69 Age					
Year	ARIMA	ARIMA with RF	ARIMA with XGB	ARIMA with SVM	ARIMA with GARCH
2022	1,140,863	1,137,779	1,131,210	1,134,584	1,156,982
2023	1,158,977	1,155,894	1,149,324	1,151,189	1,175,096
2024	1,177,092	1,174,008	1,167,439	1,168,260	1,193,211
2025	1,195,206	1,192,122	1,185,553	1,185,807	1,211,325
2026	1,213,320	1,210,236	1,203,667	1,203,799	1,229,440
2027	1,231,435	1,228,351	1,221,782	1,222,172	1,247,554
2028	1,249,549	1,246,465	1,239,896	1,240,841	1,265,669
2029	1,285,777	1,264,579	1,258,010	1,259,710	1,283,783
2030	1,303,892	1,282,694	1,276,124	1,278,690	1,301,898

**Table 9:** Different models forecasting value for 70 + Age group

70 + Age					
Year	ARIMA	ARIMA with RF	ARIMA with XGB	ARIMA with SVM	ARIMA with GRACH
2022	1,452,382	1,451,624	1,453,355	1,450,034	1,477,402
2023	1,484,539	1,483,781	1,485,512	1,481,533	1,509,560
2024	1,516,696	1,515,939	1,517,669	1,513,376	1,541,717
2025	1,548,854	1,548,096	1,549,827	1,545,512	1,573,875
2026	1,581,011	1,580,253	1,581,984	1,577,879	1,606,033
2027	1,613,168	1,612,411	1,614,141	1,610,409	1,638,190
2028	1,645,326	1,644,568	1,646,298	1,643,035	1,670,348
2029	1,677,483	1,676,725	1,678,456	1,675,701	1,702,506
2030	1,709,640	1,708,883	1,710,613	1,708,361	1,734,663

The results support the idea that death rates vary with age, and that hybrid models, especially ARIMA + SVM, are much better at predicting the future across most age groups. These findings underscore the necessity of customizing prediction models to the distinct characteristics of each group to facilitate better healthcare planning and treatments.

We assessed the calculation time for each forecasting model across all age groups to ascertain efficiency and accuracy. The independent ARIMA and hybrid ARIMA-based models demonstrated swift computation, with all execution times remaining under one second, as indicated in Table 10. Machine learning models like RF and SVM executed almost quickly (0 s); however, XGBoost had a somewhat extended computational period (up to 0.27 s). Among hybrid models, ARIMA + XGBoost often had the longest execution time (e.g., 0.47 s for the age group 0–5), while ARIMA + SVM and ARIMA + RF consistently displayed shorter execution durations. The execution duration for ARIMA + GARCH ranges from 0.1 to 0.24 s, depending on the age group. The results indicate that all models display computational efficiency and are appropriate for real-time or near-real-time applications, with only minor discrepancies in runtime.

**Table 10:** Computational Time (in seconds) for forecasting models across different Age groups

Age Group	0–5	6–15	16–49	50–69	70 +
ARIMA	0.01	0.01	0.01	0.01	0.01
ARIMA + RF	0.01	0.01	0.01	0.01	0.01
ARIMA + SVM	0.01	0.01	0.01	0.01	0.01
ARIMA + XGBoost	0.47	0.21	0.24	0.27	0.25
ARIMA + GARCH	0.12	0.14	0.17	0.22	0.24

The superior forecasting accuracy of hybrid models over a single ARIMA is crucial for public health planning. By using these estimates, policymakers may forecast the rates of cardiovascular death for specific age groups, allowing for targeted medical resource allocation, early intervention strategies, and awareness campaigns. For instance, healthcare organizations may proactively increase geriatric services, allocate intensive care units, and enhance cardiac screening programs if higher mortality is anticipated in older age groups. Additionally, integrating these forecasting models into existing healthcare systems through dashboards or automated reporting tools facilitates real-time monitoring and long-term strategic planning. To further refine intervention strategies, future developments may integrate these models with socioeconomic and environmental data.

## Conclusion and future scope

The examination of cardiovascular mortality forecasting models across various age groups revealed significant new insights into the performance of the models and the trends in illness. Hybrid models, especially ARIMA combined with SVM, often did better than standalone ARIMA for most age groups, including 6–15, 16–49, 50–69, and 70 + . It is intriguing that the ARIMA method, which is simpler, was the most appropriate for the 0–5 age range. This evidence suggests that the fundamental patterns in early life mortality may not necessitate the use of sophisticated hybrid methods. The imminent necessity of targeted treatments and the allocation of healthcare resources for the elderly is underscored by the highest death rate among the 70 + age group.

Practically speaking, these forecasting models have immense promise for use in public health planning. They can assist with early resource planning, trend tracking, and improved service delivery across multiple age groups. However, there are certain challenges; hybrid models rely heavily on data quality and volume, can be computationally intensive, and may overfit small datasets. Another difficulty is model interpretability, as public health officials want clear, practical comprehension. Future research should look into advanced deep learning approaches like LSTM, GRU, and transformer-based models, which can capture long-range temporal dependencies and hence improve real-world applicability. Including external variables, such as socioeconomic status, access to healthcare, eating habits, and environmental factors, improves model relevance and robustness even further. Incorporating uncertainty quantification would improve the reliability of policy projections. These enhancements will allow legislators to implement data-driven, age-specific policy to better address the growing burden of cardiovascular disease.

## Data Availability

Data Availability in IHME or https://www.healthdata.org/
